# Early Detection of Ovarian Cancer with Conventional and Contrast-Enhanced Transvaginal Sonography: Recent Advances and Potential Improvements

**DOI:** 10.1155/2012/302858

**Published:** 2012-04-26

**Authors:** Arthur C. Fleischer, Andrej Lyshchik, Makiko Hirari, Ryan D. Moore, Richard G. Abramson, David A. Fishman

**Affiliations:** ^1^Department of Radiology & Radiological Sciences, Vanderbilt University Medical Center, 37232 Nashville, TN, USA; ^2^Department of Obstetrics and Gynecology, Vanderbilt University Medical Center, 37232 Nashville, TN, USA; ^3^Cancer Screening Center and Cancer Institute Hospital, Japanese Foundation for Cancer Research, Tokyo, Japan; ^4^Division of Gynecologic Oncology, Mount Sinai Medical Center, 10029 New York, NY, USA

## Abstract

Recently, there have been several major technical advances in the sonographic diagnosis of ovarian cancer in its early stages. These include improved assessment of tumor morphology with transvaginal sonography (TVS), and detection and characterization of tumor neovascularity with transvaginal color Doppler sonography (TV-CDS) and contrast-enhanced transvaginal sonography (CE-TVS). This paper will discuss and illustrate these improvements and describe how they enhance detection of early-stage ovarian cancer. Our initial experience with parametric mapping of CE-TVS will also be mentioned.

## 1. Introduction

This year in the United States approximately 24,000 women will be diagnosed with ovarian cancer, and there will be approximately 14,000 associated deaths, predominantly from epithelial ovarian cancer (EOC). Worldwide, it is estimated that 204,449 patients with ovarian cancer will be diagnosed this year with an estimated 124,860 disease-related deaths. The incidence of ovarian cancer has been steadily increasing over the past 10 years, with an overall lifetime risk of 1.8% [3]. Despite improvements in surgical techniques and new chemotherapeutic regimens, the overall survival for women with stage III/IV EOC has remained relatively unchanged (15%) over the past 40 years [3]. In contrast, women diagnosed with disease confined to the ovary (stage I) require less morbid surgical intervention, may not require adjuvant chemotherapy, have a significantly improved quality of life, and most importantly have an overall 5-year survival approximating 90% [3]. Unfortunately, 75% of women continue to be diagnosed with advanced-stage disease. It is thought that accurate diagnosis of EOC at an earlier stage may decrease overall disease-related mortality.

Evaluation of adnexal masses can be performed with several imaging methods, including TVS, computed tomography (CT), magnetic resonance imaging (MRI), and positron emission tomography (PET). CT can detect large adnexal masses but has lower sensitivity for small adnexal masses, especially in thin patients in whom adnexal lesions can be misinterpreted as other pelvic structures or loops of pelvic small bowel. Furthermore, the ability of CT to characterize adnexal lesions as benign or malignant is limited by low inherent tissue contrast, with the notable exception of ovarian dermoids that can be characterized based on the presence of macroscopic fat and/or calcification. MRI offers higher spatial and contrast resolution than CT and can characterize a wider spectrum of adnexal lesions based on magnetic signal properties or enhancement behavior, but accuracy of MRI may diminish for borderline ovarian tumors and small ovarian masses. Compared to TVS, MRI is costly and has limited availability [4]. PET can identify aggressive adnexal lesions on the basis of increased fluorodeoxyglucose (FDG) uptake, but it suffers from low specificity for small lesions, noting that normal premenopausal ovaries will demonstrate increased metabolic activity at mid cycle, and a physiological corpus luteum can therefore mimic an aggressive malignancy. The accuracy of PET is also limited secondary to false negatives in borderline ovarian neoplasms [5].

TVS is widely available and offers high-resolution imaging without the use of ionizing radiation. For these reasons, TVS is the initial diagnostic modality of choice for the evaluation of most patients with a pelvic mass. As previously mentioned, TVS has limited sensitivity and specificity for the definitive diagnosis of ovarian cancer because of overlapping morphologic features seen in benign and malignant lesions. Recently, however, significant technologic advances have yielded vast improvements in the sonographic depiction of early-stage ovarian cancer, and these improvements have translated into improved sonographic discrimination of benign from malignant disease in preliminary studies. Combined evaluation of sonographic morphology and CDS forms a set of basic “simple rules” for sonographic distinction of benign from malignant ovarian masses based on the data derived from a European multicenter study which included 1,223 adnexal tumors (sensitivity 93%; specificity 90%) [6] (Table 1).

Three-dimensional transvaginal sonography (3D TVS) has improved the morphologic depiction of ovarian cancers beyond the capabilities of traditional TVS. Improvements in transvaginal color Doppler sonography (TV-CDS) have enhanced sonographic assessment of large tumor vascular networks, and contrast-enhanced transvaginal sonography (CE-TVS) now allows for interrogation of tumor microvascularity [7–9]. This paper discusses these newer techniques, specifically CE-TVS, with emphasis on their advantages and areas for potential improvement.

### 1.1. 2D and 3D TVS

Conventional sonographic criteria for ovarian cancer diagnosis are based on morphological classification of ovarian masses. Ovarian malignancy is unlikely in simple cysts with smooth walls, but presence of a solid mass or solid projections (papillary excrescences) into the cyst cavity significantly increases the risk of malignancy.

**Figure 1:**
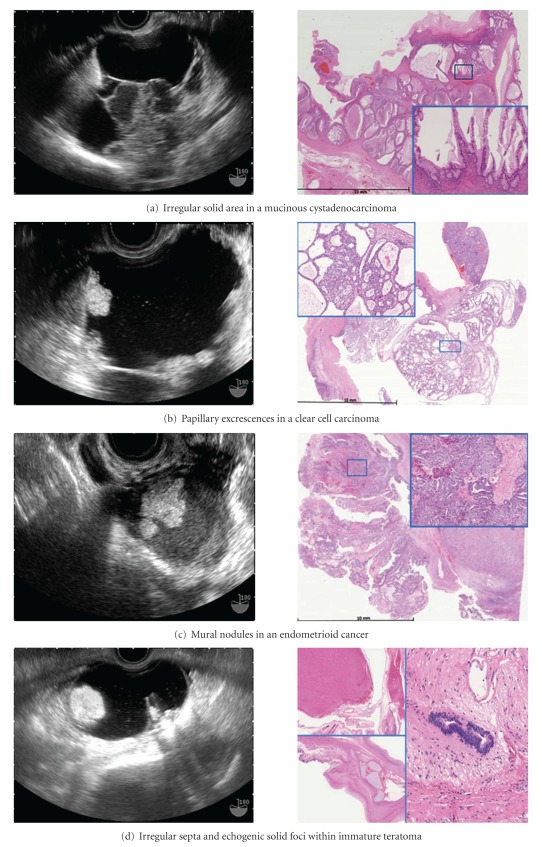
Morphologic signs of malignancy with histopathologic correlation on TVS in various histologic types of stage 1A ovarian cancer.

**Figure 2:**
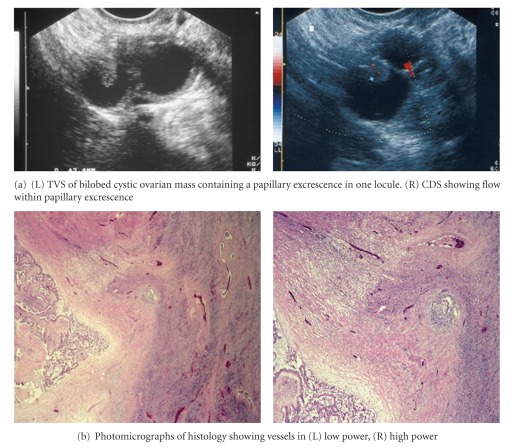
2D CDS of showing flow within papillary excrescence within a papillary cystadenofibroma.

**Figure 3:**
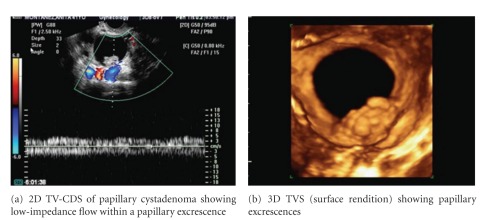
3D TV-CDS of papillary excrescences within a papillary serous cystadenoma.

Hirai has described the morphologic features on TVS associated with stage I ovarian cancer in a lay-screening population in Japan [1] (Figures 1, 2, and 3). In general, the stage IA ovarian cancers with normal CA-125 were small and had less solid components than stage IA cancers with elevated CA-125. Papillary excrescences typically occur in areas of epithelial neoplasia and can be seen borderline rather than frankly malignant lesions.

Over the last 10 years, the diagnostic accuracy for conventional 2D TVS has been improving [10]. A 1997 study reported that gray-scale sonography identified malignant tumors with a sensitivity of 91% and a specificity of 84% [11], while a 2008 study found a sensitivity of 93% and specificity of 90% [12]. As the result of these studies, several morphological scoring systems have been developed for sonography, including features such as the presence of papillary projections or irregular and/or thick septae. Results of a meta-analysis provide evidence that sonographic techniques that combine gray-scale morphologic assessment with tumor vascularity mapping are significantly better in ovarian lesion characterization than Doppler arterial resistance measurements, color Doppler flow imaging, or gray-scale morphologic information alone [13].

The recent development of 3D-TVS improves the detection of morphologic abnormalities indicative of neoplastic ovarian masses. In particular, small papillary excrescences or focal wall (mural) irregularities can be detected which are associated with epithelial malignant growth in ovarian masses [8]. The recent advent of matrix array transducers/probes may improve visualization of both internal and external wall (capsular) abnormalities, increase comfort for the patient, and increase reproducibility.

### 1.2. Transvaginal Color Doppler Sonography (TV-CDS)

TV-CDS provides depiction of the macrovascularity (over 200 *μ*) of tumors but does not delineate microscopic (capillary) tumor neovascularity. The vascular network in tumors can be further interrogated using Doppler techniques to indicate the impedance within vessels [7, 8]. This in turn roughly reflects pressure gradients.

Combining morphologic assessment with TVS with color Doppler features has allowed accurate assessments of whether a mass is benign or malignant by following “simple rules” [6]. Using color Doppler techniques, the overall vascularity was classified as high, low, or intermediate, rather than determining vascular indices such as resistance or pulsatility. In a European multicentered study, it was shown that this paradigm resulted in 90% sensitivity and 92% sensitivity [6].

### 1.3. Contrast-Enhanced Transvaginal Sonography (CE-TVS)

**Figure 4:**
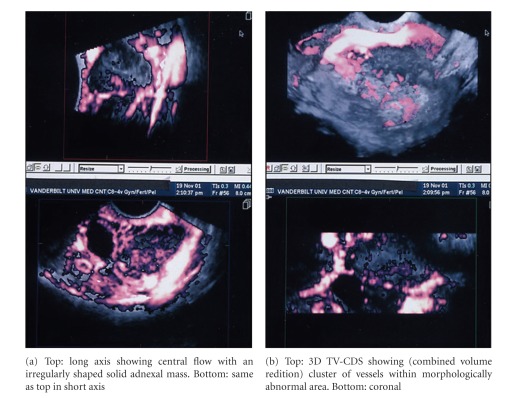
3D TV-CDS of papillary cystadenocarcinoma showing multiplanar reconstruction (MPR) images.

**Figure 5:**
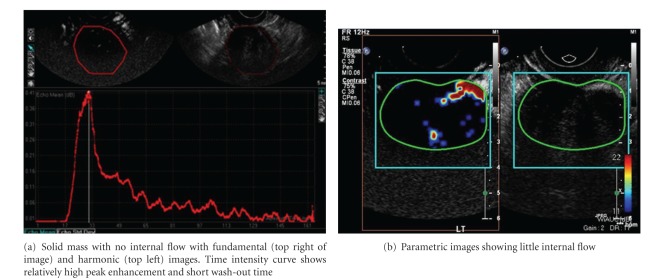
CE-TVS of a benign fibroma.

**Figure 6:**
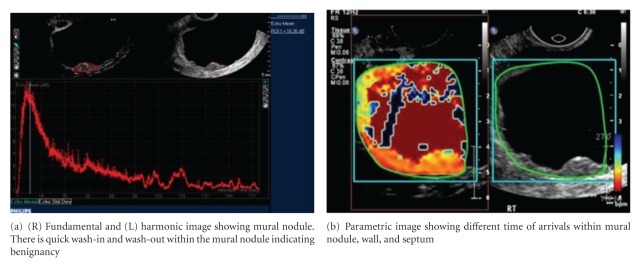
CE-TVS of serous cystadenoma with mural nodules.

Both micro- and macroscopically, tumor neovascularity is characterized as vessels that demonstrate irregular caliber and branching. TV-CDS can only detect flow in relatively large vessels. Microvascular (i.e., capillary) tumor neovascularity can be depicted using microbubble contrast (Figures 4, 5, and 6). On dynamic CE-TVS malignant tumor neovascularity usually demonstrates a higher peak of contrast enhancement and prolonged contrast washout when compared to benign tumors [1, 9, 14, 15] (Figure 9). 

In our previous study, all malignant tumors and 50% of benign tumors showed detectable contrast enhancement (image intensity > 10% above the baseline) after microbubble injection [9]. When contrast enhancement dynamics were assessed, we found that malignant lesions had a similar time to peak (*T*_p_; 26.2 ± 5.9 versus 29.8 ± 13.4 seconds; *P* = .4), greater peak enhancement (PE; 21.3 ± 4.7 versus 8.3 ± 5.7 dB; *P* < .001), a longer half wash-out time ((^1^/_2_)*T*_wo_; 104.2 ± 48.1 versus 32.2 ± 18.9 seconds; *P* < .001), and a greater area under the curve (AUC; 1807.2 ± 588.3 versus 413.8 ± 294.8 seconds^−1^; *P* < .001) when compared with enhancing benign lesions (Figure 9).

AUC greater than 787 seconds^−1^ was the most accurate diagnostic criterion for ovarian cancer, with 100.0% sensitivity and 96.2% specificity. Additionally, PE greater than 17.2 dB (90.0% sensitivity and 98.3% specificity) and a (1/2)*T*_wo_ of greater than 41.0 seconds (100.0% sensitivity and 92.3% specificity) proved to be useful. Initial analysis of contrast-enhanced kinetic was done using time intensity curves for mean, standard deviation, and *P* value (Figures 9 and 11) and subsequently by receive operator characteristic curves for vascular index (VI); flow index (FI); vascular flow index (VFI) (Figure 12). The receiver operator characteristics of each parameter is shown in Figure 13 with predetermined cutoff values as established with receive operation curves and compared to VI, FI, and VFI values. These results show that contrast-enhanced nonlinear pulse inversion sonography is a more appropriate method for characterizing blood flow dynamics in ovarian tumors than by TV-CDS and can provide an important tool to aid differential diagnoses between benign and malignant ovarian tumors.

### 1.4. Parametric Mapping of CE-TVS

While the time-intensity curve has traditionally been calculated from mean signal intensities over a region of interest, parametric mapping of time-intensity curve variables on a pixel-by-pixel basis allows for more global visualization of tumor hemodynamics. The use of this technique in ovarian cancer has been limited to selecting the pixel with greatest peak enhancement (PE) and using that pixel's time-intensity curve for further analysis [15]. However, parametric maps of CE-TVS have recently been used with limited success for differentiation of benign and malignant focal liver lesions [16] and breast lesions [17, 18]. Preliminary results for using parametric mapping of ovarian tumors seem to indicate significant potential for improving diagnostic accuracy.

Our preliminary results from a subset of 29 out of the 57 subjects analyzed in our previous region of interest (ROI) study show potential for this technique to differentiate benign and malignant ovarian masses [19]. The methods of data acquisition are outlined in the previously described study [9]. Analysis with a quantification software prototype (Bracco Suisse SA, Geneva, Switzerland) utilized parametric maps of *T*_p_ (sec), PE (dB), and wash-in AUC (wiAUC; arbitrary units, a.u.). The region of interest was kept constant in size between subjects and was corrected for motion. The map color scales were adjusted such that abnormal hemodynamics were represented by red for PE > 24 a.u, *T*_p_ < 11 *s*, and wiAUC > 35 a.u. (cutoffs chosen at optimum points on receiver operator characteristics curve), and the presence of any red color was used to differentiate benign and malignant tumors.

The preliminary results from the subanalysis of 18 benign and 11 histologically proven malignant ovarian masses showed greatest diagnostic accuracy for maps of PE (sensitivity 100%, specificity 67%) and wiAUC (sensitivity 73%, specificity 94%), while maps of *T*_p_ were least accurate (sensitivity 100%, specificity 17%). Final analysis of all 57 subjects is needed to determine the ultimate utility of these methods, but preliminary results are promising.

## 2. Discussion

CE-TVS can significantly improve the diagnostic ability of transvaginal sonography alone to identify early microvascular changes that are known to be associated with early-stage ovarian cancer [1, 9, 14, 15, 20]. Currently, contrast agents play a pivotal role in the imaging modalities of CT and MRI by increasing lesion conspicuity, accentuating morphologic features within a lesion, and defining time-resolved lesion enhancement patterns that serve as additional imaging parameters by which a lesion may be characterized. Indeed, contrast agents have received such widespread acceptance that a CT exam performed without intravenous contrast or an MRI without contrast for many indications is now considered limited. Preclinical studies demonstrated that the intravenous contrast agents for sonography hold great promise in a multitude of potential clinical applications, especially in identifying aberrant vascular changes associated with malignancy [19, 21].

Previous studies have addressed the use of CE-TVS for benign and malignant tumors by showing greater enhancement of malignant tumors on Doppler imaging. According to the initial work reported by Kupesic and Kurjak, the use of a contrast agent with 3D power Doppler sonography showed very high diagnostic efficiency (95.6%) that was superior to that of nonenhanced 3D power Doppler sonography (86.7%) [22]. However, simple documentation of tumor enhancement may not be sufficient because some benign tumors show detectable contrast enhancement. This limitation can be addressed by assessment of the contrast enhancement kinetics. Only a few studies have been published that used kinetic parameters of the contrast agent to compare benign with malignant tumors in the power Doppler mode. Orden et al. demonstrated that after microbubble contrast agent injection, malignant and benign adnexal lesions behave differently in degree, onset, and duration of Doppler US enhancement. Doppler contrast-enhanced parameters in that study had 79–100% sensitivity and 77–92% specificity [23]. Marret et al. reported that wash-out times and AUC were significantly greater in ovarian malignancies than in other benign tumors (*P* < .001), leading to sensitivity estimates between 96% and 100% and specificity estimates between 83 and 98% [14]. They concluded that Doppler contrast-enhanced parameters had slightly higher sensitivity and slightly lower specificity when compared with transvaginal sonographic variables of the resistive index and serum CA-125 levels [15]. 

Our preliminary clinical studies explored differences in enhancement parameters in benign versus malignant ovarian masses using a new method of CE-TVS termed pulse inversion nonlinear imaging [9]. This method produces more reliable estimates of tumor microvascular perfusion and provides more consistent results compared to Doppler-based contrast-enhanced ultrasound. Our data suggest that, except for the *T*_p_, contrast enhancement parameters are significantly different in benign versus malignant ovarian masses. The *T*_p_ probably reflects intrinsic circulation depending on cardiac contraction, blood pressure, and overall vascular tone. Once blood circulates through the tumor, however, differences may reflect the unique branching patterns and vessel morphologic characteristics in the microvascularity of the tumors.

As a general statement, contrast enhancement patterns significantly differ between benign and malignant ovarian masses. The addition of a vascular sonographic contrast agent allows a more complete delineation of the vascular anatomy through enhancement of the signal strength from small vessels (capillaries) and provides an entirely new opportunity to time the transit of an injected bolus. CE-TVS has higher sensitivity and specificity to differentiate between benign and malignant lesions than conventional TVS and for detecting occult stage I disease.

### 2.1. Future Improvements in CE-TVS

**Figure 7:**
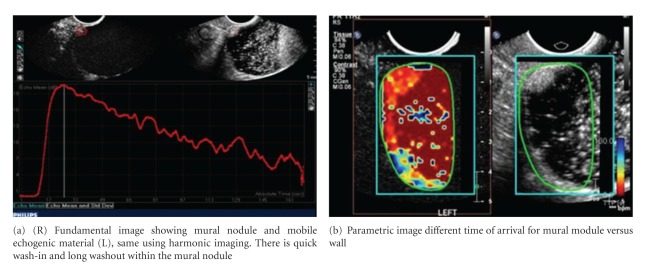
CE-TVS of borderline mucinous (intestinal) cystadenocarcinoma.

**Figure 8:**
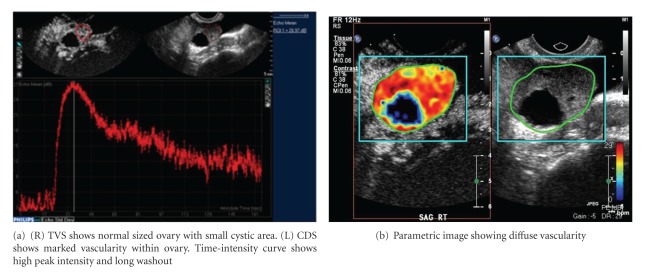
CE-TVS of stage I papillary serous cystadenocarcinoma.

**Figure 9:**
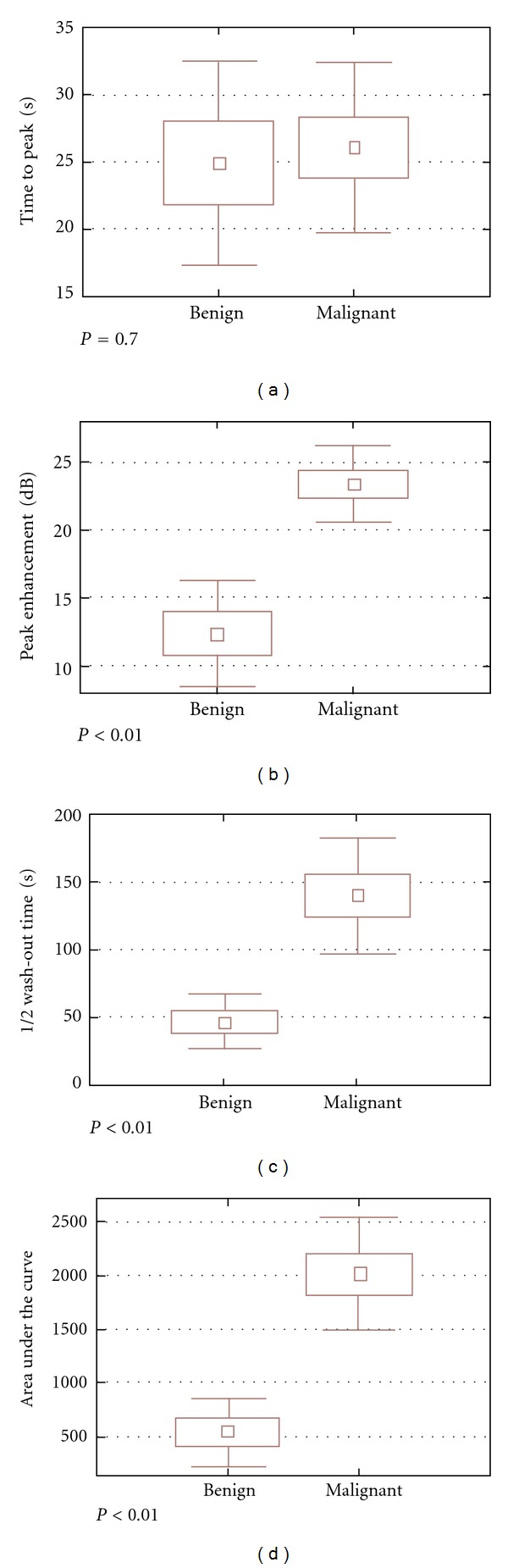
Box graph of contrast-enhanced parameters. While there is no difference in time of peak (*T* wash-in), there are significant differences in peak enhancement, wash-out time and vascularity ((b), (c); (d)) from [1].

**Figure 11:**
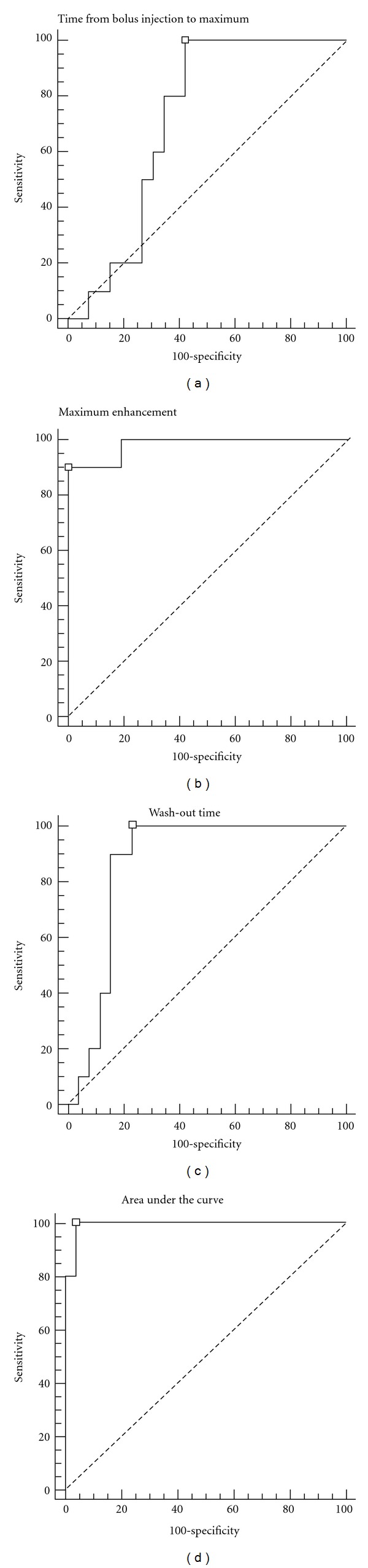
Receiver operator characteristerics for (a) wash-in, (b) maximum enhancement, (c) wash-out, and (d) area under curve.

**Figure 12:**
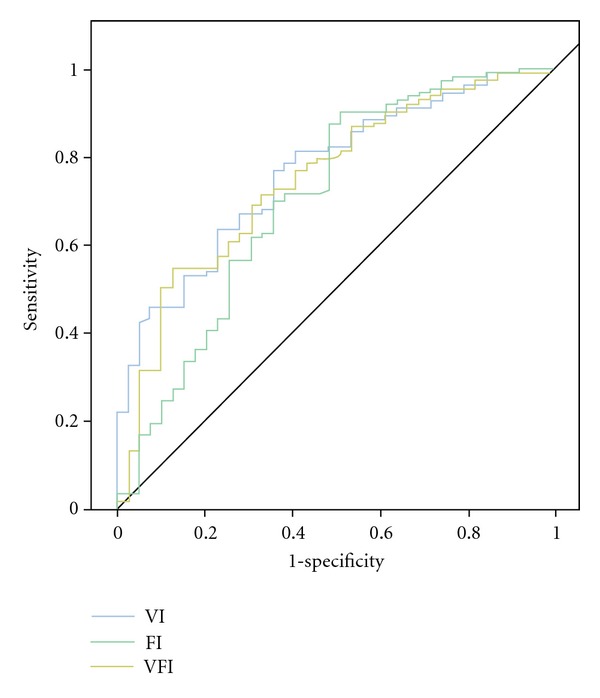
Receiver operator characteristic of various parameters showing cutoff points for vascular index (VI), flow index (FI), and vascular flow index (VFI) (from [2]).

**Figure 13:**
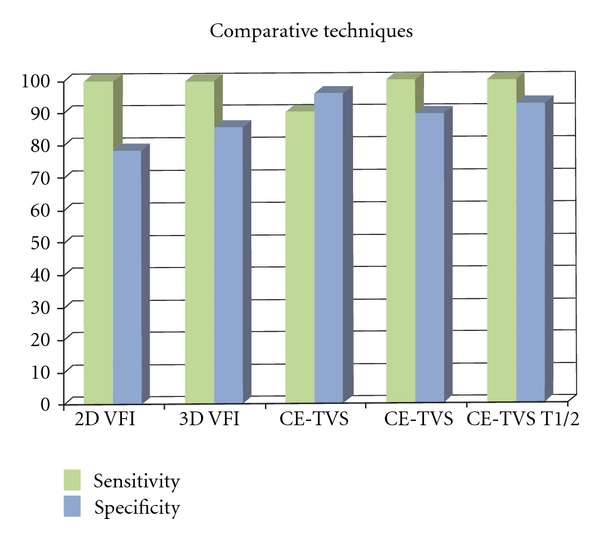
Relative accuracy (sensitivity and specificity of enhancement kinetic parameters) of various techniques using predetermined cutoff points of: 2D VFI (>0.4), 3D VFI (>0.5), CE-TVS (max >17.2 dB), CE-TVS ((1/2)*T*_wo_ > 41 sec), CE-TVS (AUC > 787 s^−1^).

CE-TVS can detect tumor neovascularity (Figures 4, 5, 6, 7, and 8). Tumor neovascularity is characterized by vessels with abnormal endothelial structure that are irregular in caliber and branching patterns. In order to recognize these features, contrast enhancement kinetics show relatively high vascular volume (AUC) and PE. These parameters seem to be best depicted using time-intensity curves. These parameters may also be shown in a parametric map, which allows for stricter cutoff criterion than ROI analysis, as peak values are visualized on a much finer scale than typical ROIs. The traditional approach of calculating the time-intensity curve over ROIs chosen on morphology alone allows small areas of neovascularity to be missed, as they are averaged over larger heterogeneous areas. The primary utility of parametric mapping of ovarian tumors may be guiding selection of ROIs to the area of greatest malignant potential.

With improved imaging technology comes the potential for enhanced therapeutic measures. Specifically, this includes directed therapeutic measures after labeled microbubbles are used [2]. In a murine model this method has been shown to accurately detect sites of active angiogenesis [24]. It is possible that labeled microbubbles could provide directed therapy. 

In conclusion, it is hoped that this paper may contribute to the development of new methods for diagnosing, enhancing therapy, and detecting tumor response for this dreaded gynecologic malignancy, possibly with the use of targeted microbubbles [2, 24–28].

## Figures and Tables

**Figure 10:**
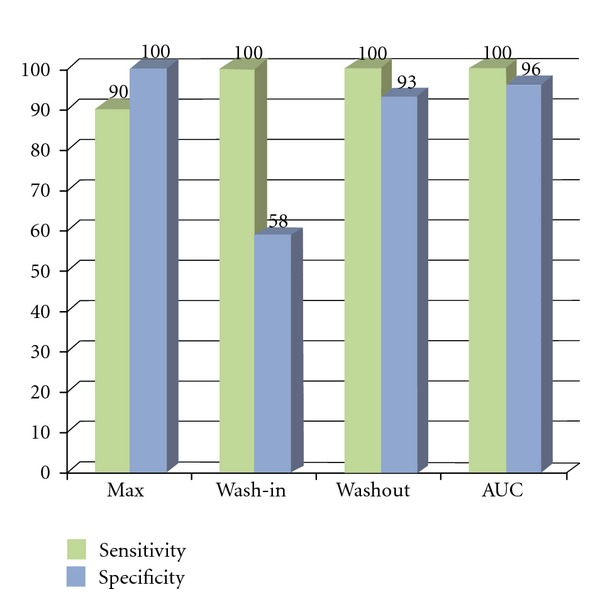
Sensitivities and specificities of maximum enhancement, wash-in, wash-out and area under curve (AUC). Maximum enhancement, wash-out and AUC had greatest accuracy.

**Table 1:** “Simple rules” for sonographic diagnosis of ovarian cancer*.

Benign	Malignant
(1) Unilocular cyst	(1) Irregular solid tumor
(2) Solid components <7 mm	(2) Ascites
(3) Acoustic shadows	(3) Papillary excrescences
(4) Smooth multilocular	(4) Irregular multiloculated/solid tumor
<10 cm	>10 cm
(5) No color Doppler flow	(5) Very high color content

*Timmerman, D, US O/G 31 : 681, 2008.
